# Recombinant human activated protein C resets thrombin generation in patients with severe sepsis – a case control study

**DOI:** 10.1186/cc3774

**Published:** 2005-07-21

**Authors:** Anne-Cornélie JM de Pont, Kamran Bakhtiari, Barbara A Hutten, Evert de Jonge, Margreeth B Vroom, Joost CM Meijers, Harry R Büller, Marcel Levi

**Affiliations:** 1 Intensivist, Department of Intensive Care, Academic Medical Center, University of Amsterdam, The Netherlands; 2 Laboratory Researcher, Department of Vascular Medicine, Academic Medical Center, University of Amsterdam, The Netherlands; 3 Clinical Epidemiologist, Department of Epidemiology and Biostatistics, Academic Medical Center, University of Amsterdam, The Netherlands; 4 Professor of Intensive Care Medicine, Department of Intensive Care, Academic Medical Center, University of Amsterdam, The Netherlands; 5 Head of the Laboratory of Vascular Medicine, Department of Vascular Medicine, Academic Medical Center, University of Amsterdam, The Netherlands; 6 Professor of Vascular Medicine, Department of Vascular Medicine, Academic Medical Center, University of Amsterdam, The Netherlands; 7 Professor of Internal Medicine, Department of Internal Medicine, Academic Medical Center, University of Amsterdam, The Netherlands

## Abstract

**Introduction:**

Recombinant human activated protein C (rhAPC) is the first drug for which a reduction of mortality in severe sepsis has been demonstrated. However, the mechanism by which this reduction in mortality is achieved is still not clearly defined. The aim of the present study was to evaluate the dynamics of the anticoagulant, anti-inflammatory and pro-fibrinolytic action of rhAPC in patients with severe sepsis, by comparing rhAPC-treated patients with case controls.

**Methods:**

In this prospectively designed multicenter case control study, 12 patients who were participating in the ENHANCE study, an open-label study of rhAPC in severe sepsis, were treated intravenously with rhAPC at a constant rate of 24 μg/kg/h for a total of 96 h. Twelve controls with severe sepsis matching the inclusion criteria received standard therapy. The treatment was started within 48 h after the onset of organ failure. Blood samples were taken before the start of the infusion and at 4, 8, 24, 48, 96 and 168 h, for determination of parameters of coagulation and inflammation.

**Results:**

Sepsis-induced thrombin generation as measured by thrombin-antithrombin complexes and prothrombin fragment F1+2, was reset by rhAPC within the first 8 h of infusion. The administration of rhAPC did not influence parameters of fibrinolysis and inflammation. There was no difference in outcome or occurrence of serious adverse events between the treatment group and the control group.

**Conclusion:**

Sepsis-induced thrombin generation in severely septic patients is reset by rhAPC within the first 8 h of infusion without influencing parameters of fibrinolysis and inflammation.

## Introduction

During severe sepsis, activation of the inflammatory cascade leads to cell damage and organ failure. In recent years, the importance of the cross-talk between coagulation and inflammation in severe sepsis has been well defined. This has led to the hypothesis that inhibitors of coagulation might have a dual effect, that is, interruption of the cascades of both coagulation and inflammation. Recombinant human activated protein C (rhAPC, drotrecogin alfa (activated), Xigris^®^) is the first drug for which a reduction of mortality in severe sepsis has been demonstrated [1]. Indeed, rhAPC is an effective anticoagulant and also has distinct anti-inflammatory effects, at least *in vitro*. However, the mechanism by which the reduction in mortality by rhAPC is achieved is still not clearly defined. Several mechanisms have been proposed. Firstly, rhAPC may inhibit the formation of thrombin by proteolytically degrading coagulation factors Va and VIIIa. Thrombin has a central role in coagulation due to its ability to convert fibrinogen to fibrin, but also as the most potent agonist of platelet activation [2]. Thrombin may also affect the production of inflammatory cytokines by binding to protease-activated receptors (PARs) in mononuclear cells [3]. Secondly, rhAPC may inhibit the action of plasminogen activator inhibitor type I (PAI-I), thereby restoring the suppressed fibrinolytic state during sepsis [4]. Thirdly, binding of rhAPC to the endothelial protein C receptor (EPCR) may influence gene expression profiles of cells by blocking nuclear factor kappa B nuclear translocation, which is required for increases in proinflammatory cytokines and adhesion molecules [5]. Direct activation of PAR-1 by the APC-EPCR complex is another mechanism by which APC may affect inflammation [6]. Fourthly, it is hypothesized that rhAPC inhibits the adherence of activated leukocytes to activated endothelium [5,7]. However, the relative importance of each of these mechanisms for the mortality reduction in severe sepsis by rhAPC is still unclear.

The aim of the current study was to evaluate the dynamics of the anticoagulant, anti-inflammatory and pro-fibrinolytic action of rhAPC in patients with severe sepsis, by comparing rhAPC-treated patients with case controls. For this purpose, we employed sensitive assays for the assessment of activation and inhibition of the coagulant, inflammatory and fibrinolytic system.

## Methods

### Study design

The ENHANCE study, an open-label study of rhAPC in severe sepsis, was conducted worldwide and more than 2000 patients were included. In the Netherlands, four sites participated in the current ENHANCE substudy, three academic hospitals and one large teaching hospital. After completion of the ENHANCE study, an equal number of patients with severe sepsis meeting identical inclusion criteria were prospectively enrolled in this substudy to serve as case controls. At the time of performance of the study, rhAPC was not yet licensed and available for routine treatment of patients with severe sepsis.

### Patients

The study was approved by the institutional review board and written informed consent was obtained from all participants or their authorized representatives. Patients were eligible for the trial if they had a known or suspected infection, three or more signs of systemic inflammation and a sepsis-induced dysfunction of at least one organ system that lasted no longer than 48 h. In patients enrolled in the ENHANCE study, treatment with rhAPC was started within 48 h after they met the inclusion criteria. The time of starting the rhAPC infusion was considered as t = 0. In the controls, the time at which rhAPC would have been started if the patient had been in the treatment group, was considered as t = 0. Blood samples were taken at the same time points in both the treatment group and the control group.

### Treatment

In the treatment group, rhAPC was administered intravenously at a constant rate of 24 μg/kg body weight per hour for a total duration of 96 h. The infusion was interrupted for 1 h before any percutaneous procedure and was resumed 1 h later. During the infusion of rhAPC, no other anticoagulant was administered except nadroparin or dalteparin in a prophylactic dose. Except for the administration of rhAPC, the treatment of patients in both groups was identical.

### Blood collection

Platelet counts were routinely determined daily. Blood for the analysis of parameters of coagulation and inflammation was collected from an arterial line just before t = 0 and at 4, 8, 24, 48, 96 and 168 h. Blood for platelet counts and cytokine assays was collected in K3-EDTA-containing tubes. All other blood samples were collected in citrated vacutainer tubes. Plasma was prepared by centrifugation of blood at 2500 × *g *twice for 20 mins at 16°C, followed by storage at -80°C until assays were performed.

### Laboratory assays

The plasma concentrations of thrombin-antithrombin complexes (TAT), prothrombin fragment F1+2 (F1+2), and plasmin-antiplasmin complexes (PAP) were measured by ELISA (Dade Behring, Marburg, Germany). Activated partial thromboplastin time (APTT) and prothrombin time (PT) were performed on an automated coagulation analyzer (Behring Coagulation System, BCS) with reagents and protocols from the manufacturer (Dade Behring). Protein C was determined using the Coamatic protein C activity kit from Chromogenix (Milan, Italy). Total protein S antigen was assayed by ELISA using antibodies from DAKO (Glostrup, Denmark). Protein C and S deficiencies were defined as activity levels of <65% of the level measured in normal pool plasma. Free protein S was measured by precipitating the C4b-binding protein-bound fraction with polyethylene glycol 8000 and measuring the concentration of free protein S in the supernatant. Free protein S deficiency was defined as an activity of <26% of the total protein S level. Protein C inhibitor (PCI) was determined by ELISA as described by Elisen *et al*. [8]. Normal PCI levels vary from 55 to 142% of the PCI level measured in normal pool plasma. Thrombin-activatable fibrinolysis inhibitor (TAFI) antigen levels were assayed by ELISA as described by Mosnier *et al*. [9]. Normal TAFI levels vary from 50 to 150% of the TAFI level measured in normal pool plasma. D-dimers were assayed with an ELISA (Asserachrom D-Dimer, Roche, Almere, the Netherlands). Platelet counts were assessed by a Cell-dyn 4000 analyzer (Abbott Laboratories, Abbott Park, IL, USA). Tumor necrosis factor alpha (TNF-alpha), interleukin (IL)-6, and IL-10 were measured using commercial ELISA kits (Central Laboratory of the Netherlands Red Cross Blood Transfusion Service, Amsterdam, the Netherlands). The assay detection limits were 3 pg/ml for TNF-alpha, 0.6 pg/ml for IL-6 and 1.2 pg/ml for IL-10. All assays were performed by the Laboratory for Clinical Chemistry, Hematology and Transfusion and the Laboratory of Experimental Vascular Medicine at the Academic Medical Center Amsterdam, the Netherlands.

### Evaluation of patients

Patients were followed for 28 days after enrollment or until death. Baseline characteristics were assessed within 24 h prior to enrollment. Microbiologic culture results were assessed daily from enrollment through day 28.

### Statistical analysis

Data were analyzed using SPSS for Windows, v11.0 (SPSS Inc, Chicago, IL, USA). Differences between the treatment group and the case control group were tested by analysis of repeated measures using mixed linear models. Changes in time within the same group were analyzed by 1-way analysis of variance. Values are given as means ± SD. Significance was defined as *p *< 0.05.

## Results

### Patient characteristics

During the ENHANCE study, 12 patients were enrolled in the substudy at the four participating sites. Another 12 patients with severe sepsis were enrolled prospectively as case controls at two of the four participating sites. The baseline characteristics of the two patient groups are shown in Table 1. There were more patients with malignancy in the control group, but all other baseline characteristics were similar. The lung was the most common site of infection in both groups and Gram-negative infections were most common. The time elapsed between meeting the inclusion criteria and t = 0 was 12.3 ± 13.2 h in the rhAPC group versus 26.7 ± 12.5 h in the control group (*p *= 0.01).

### Thrombin generation

**Figure 1:**
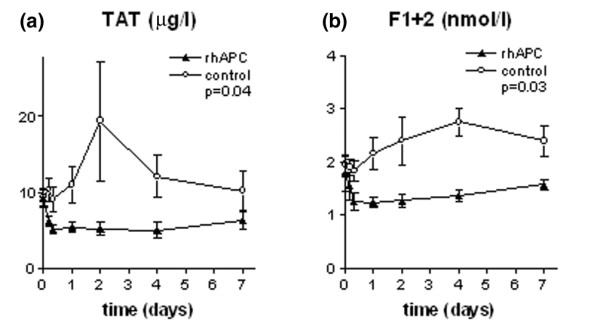
Levels of TAT and F1+2. Plasma levels of **(a) **TAT and **(b) **F1+2 in the rhAPC group (▴) and the control group (○). Data represent mean ± SD. F1+2, prothrombin fragment F1+2; rhAPC, recombinant human activated protein C; TAT, thrombin-antithrombin complexes.

Administration of rhAPC resulted in a reduction of sepsis-induced thrombin generation, as reflected by a decrease in the levels of TAT and F1+2 to 45 and 30% below baseline, respectively, within 8 h, without a significant change for the remaining 7 days (Fig. 1). In the control group, TAT and F1+2 levels increased to 2 and 1.4 times baseline, respectively, within 4 days. The difference in F1+2 levels between the two groups reached significance after 8 h (*p *= 0.03) and remained significant for the remaining 7 days (*p *= 0.03). In the rhAPC group, the APTT rose to a maximum of 1.4 times baseline within 4 h after the start of the infusion (*p *= 0.004), whereas the APTT remained stable in the control group. In both treatment groups, the PT decreased over time. However, this decrease only reached statistical significance in the control group on day 4 and day 7 (data not shown).

### Protein C pathway

**Figure 2:**
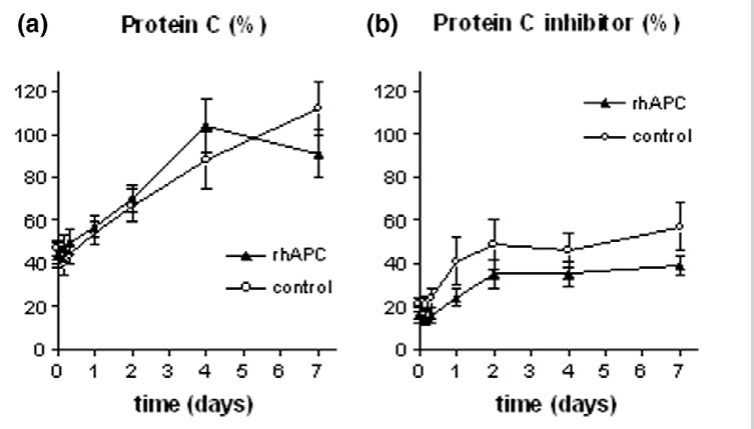
Levels of protein C and protein C inhibitor. Plasma levels of **(a) **protein C and **(b) **protein C inhibitor in the rhAPC group (▴) and the control group (○). The levels of protein C and protein C inhibitor are expressed as the percentage of the level measured in normal pool plasma. Data represent mean ± SD. rhAPC, recombinant human activated protein C.

Ninety-two percent of all patients were protein C deficient at baseline, with mean protein C levels of 44 ± 20% in the rhAPC group and 47 ± 12% in the control group (NS). As shown in Fig. 2, protein C levels normalized in the course of 2 days in both treatment groups. All patients were deficient in protein C inhibitor with mean levels of 16 ± 13% in the rhAPC group and 21 ± 11% in the control group (NS). The levels of protein C inhibitor more than doubled over time in both groups (*p *= 0.004). The increase was more pronounced in the control group, but the difference between groups did not reach statistical significance (Fig. 2).

**Figure 3:**
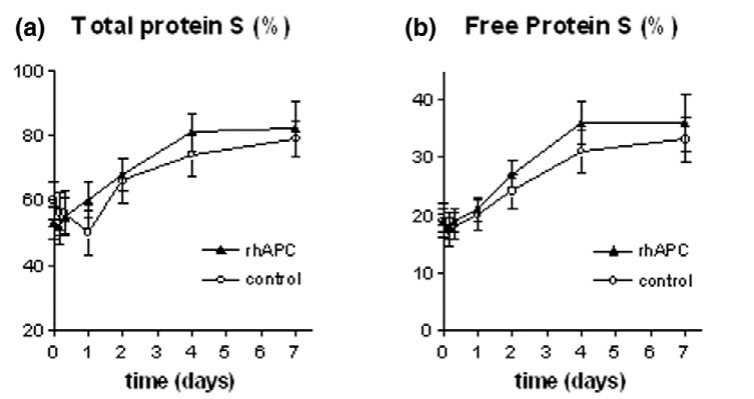
Levels of total and free protein S. Plasma levels of **(a) **total protein S and **(b) **free protein S in the rhAPC group (▴) and the control group (○). The levels of total protein S are expressed as the percentage of the level measured in normal pool plasma. The levels of free protein S are expressed as a percentage of total protein S. Data represent mean ± SD. rhAPC, recombinant human activated protein C.

At baseline, deficiency in total and free protein S was present in 63 and 79% of all patients, respectively. Mean total and free protein S levels in the rhAPC group were 53 ± 17% and 19 ± 7%, respectively, and in the control group 60 ± 20% and 19 ± 10%, respectively (NS). The levels of total and free protein S normalized in the course of 2 and 4 days, respectively (Fig. 3).

### Platelet counts

Baseline platelet counts were 173 ± 121 × 10^9^/l in the rhAPC group and 156 ± 85 × 10^9^/l in the control group (NS). In the rhAPC group, there was a trend toward an increase in platelet count: platelets increased from 173 ± 121 to 270 ± 190 × 10^9^/l on day 6 (*p *= 0.06). In the control group, the platelet count increased from 156 ± 85 to 202 ± 189 × 10^9^/l on day 6 (*p *= 0.44). The difference between the two groups was too small to reach statistical significance.

### Fibrinolysis

**Figure 4:**
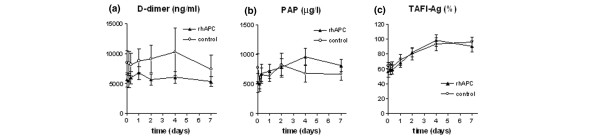
Fibrinolysis. Plasma levels of **(a) **D-dimer, **(b) **PAP and **(c)**TAFI-Ag in the rhAPC group (▴) and the control group (○). TAFI levels are expressed as the percentage of the level measured in normal pool plasma. Data represent mean ± SD. PAP, plasmin-antiplasmin complexes; TAFI-Ag, thrombin-activatable fibrinolysis inhibitor antigen; rhAPC, recombinant human activated protein C.

Parameters of fibrinolysis are shown in Fig. 4. Baseline D-dimer levels did not differ significantly between groups and did not change significantly over time. PAP levels tended to increase in the rhAPC group, whereas they remained stable in the control group. However, the difference between groups was too small to reach statistical significance. In both groups, TAFI levels were depressed at baseline (56 ± 26% in the rhAPC group and 64 ± 16% in the control group), returning to normal in the course of 4 days, without a significant difference between groups.

### Cytokines

**Figure 5:**
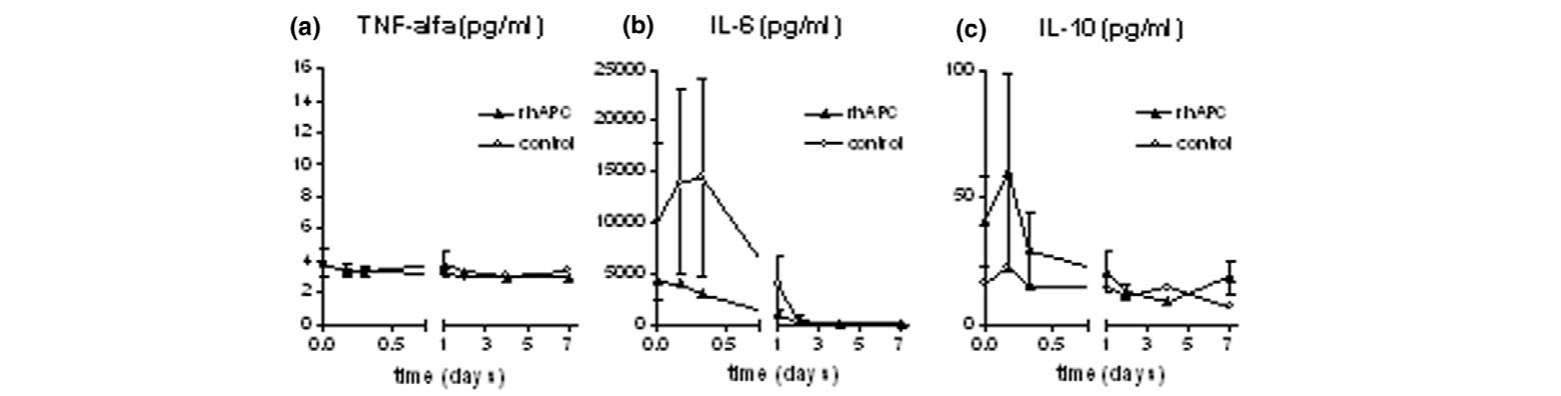
Cytokines. Plasma levels of **(a) **TNF-alpha, **(b) **IL-6 and **(c) **IL-10 in the rhAPC group (▴) and the control group (○). Assay detection limits were 3.0 pg/ml for TNF-alpha, 0.6 pg/ml for IL-6 and 1.2 pg/ml for IL-10. Data represent mean ± SD. IL, interleukin; rhAPC, recombinant human activated protein C; TNF, tumor necrosis factor.

The time course of cytokine levels is depicted in Fig. 5. Levels of TNF-alpha remained stable in both the rhAPC group and the control group. Levels of IL-6 and IL-10 gradually decreased over time in both groups, without a significant difference between groups.

### Outcome

The outcome of patients in both groups is summarized in Table 2. In total, five patients died within 28 days, two in the rhAPC group and three in the control group, which comes down to a 28-day mortality rate of 17 and 25%, respectively (*p *= 1.0). In this small sample of patients, there was no statistically significant difference between the rhAPC group and the control group with respect to length of ICU stay, length of hospital stay and percentage of patients discharged home.

### Complications

The occurrence of adverse events is summarized in Table 2. A serious bleeding event occurred in only one patient in the rhAPC group. In this patient, a central venous line was inserted erroneously without stopping the rhAPC infusion. The subsequent ongoing bleeding from the puncture site ultimately required a red blood cell (RBC) transfusion. Blood transfusion requirements were similar in the rhAPC group and the control group (0.44 ± 0.53 versus 0.23 ± 0.35 RBC units per day, respectively, *p *= 0.27).

## Discussion

In the present clinical study, we studied the dynamics of the anticoagulant, pro-fibrinolytic and anti-inflammatory action of rhAPC when used in severe sepsis, by comparing rhAPC treated patients with case controls. We demonstrated that sepsis-induced thrombin generation was reset by rhAPC, as reflected by a decrease in TAT and F1+2 levels within 8 h of infusion. We did not find any influence of rhAPC on parameters of fibrinolysis and inflammation. Although the delay between meeting the inclusion criteria and t = 0 was longer in the control group, we do not think that this difference has influenced our results. Indeed, shifting the control group curves in Figs 1, 2, 3, 4, 5 to the right for 12 h did not change the results of the comparison between the two treatment groups.

The inhibition of thrombin generation by rhAPC might be the main mechanism by which mortality reduction in patients with severe sepsis was achieved in the PROWESS study [1]. Mortality in severe sepsis is usually due to multiple organ failure, which is believed to be caused by microvascular thrombosis, impairing the blood supply to various organs [10,11]. Under physiological circumstances, thrombin generation is regulated by the protein C system in order to prevent microvascular thrombosis. During sepsis, however, the expression of thrombomodulin and EPCR on the endothelial cell surface is downregulated, leading to inadequate activation of protein C and thus to inadequate inhibition of thrombin generation.

Our findings confirm the results of Dhainaut *et al*., who demonstrated that treatment with rhAPC attenuates thrombin generation, as reflected by a significant inhibition of TAT and F1+2 [12]. In our study, the inhibition was even more pronounced: treatment with rhAPC prevented the increase in thrombin generation that occurred in the control group. Interestingly, TAT and F1+2 levels did not change from 8 h until 7 days after starting the treatment, even after stopping the rhAPC infusion. These results are in contrast with those of Dhainaut *et al*., who found an increase in levels of TAT and F1+2 on day 5. There are several possible explanations for this difference. Firstly, we did not take measurements on days 5 and 6 and might have missed a transient rise in thrombin generation. Secondly, the rhAPC group in the PROWESS study might have been more severely ill at inclusion, as the mean APACHE II score was higher than in our rhAPC group (24.6 ± 7.6 versus 21 ± 6). It is conceivable that in more severely ill patients, normalization of thrombin generation takes more time. Thirdly, the time from inclusion to drug infusion was 17.5 ± 12.8 h in the PROWESS study, as compared with 12.3 ± 13.2 h in our study. It is also conceivable that the shorter delay to treatment might have influenced the speed of recovery. If rhAPC is indeed able to reset thrombin generation within 8 h in less severely ill patients when treated within 12 h of admission, one could argue that, under these circumstances, a shorter duration of rhAPC infusion might be sufficient to achieve the same extent of inhibition of thrombin generation. This could have important consequences for the recommended duration of treatment. However, based on the results of the present study, one cannot conclude that limitation of the duration of rhAPC treatment would yield the same results. Additional studies are needed to determine under which circumstances the duration of rhAPC infusion can be limited without influencing efficacy.

At baseline, 92% of our septic patients were protein C deficient with a mean protein C level of 45.8%. This finding is consistent with the results of earlier studies. Boldt *et al*. found a baseline protein C level of 47.8% in septic patients [13] and in the PROWESS study, Bernard *et al*. found median baseline protein C levels of 47 and 50% in the rhAPC group and the control group, respectively [1]. The depletion of protein C during sepsis is caused by a combination of degradation of protein C by neutrophil elastase and inadequate biosynthesis in the liver [11,14]. In our study, the protein C levels returned to normal in the course of 2 days in both treatment groups, whereas in the study by Dhainaut *et al*., normalization of protein C levels took 3.5 days in the rhAPC group and 5 days in the control group [12]. The increased time needed for the normalization of protein C levels might reflect the greater severity of illness of patients in this study.

In the present study, we did not find a convincing effect of the administration of rhAPC on fibrinolysis. The levels of D-dimers remained unchanged over time in both the rhAPC group and the control group. This is in contrast with the findings of Bernard *et al*., who found a significant decrease in D-dimer levels in the rhAPC group as compared with the control group [1]. The fact that we did not find such an effect may be due to the small number of patients and the great interpatient variability in D-dimer levels. PAP levels showed a tendency to increase in the rhAPC group, but this increase was too small to reach statistical significance. In agreement with our findings, Dhainaut *et al*. did not find an effect of rhAPC on PAI-1, a marker of fibrinolysis, when they used the method of repeated measurements [12]. They concluded that their results do not provide a strong basis for a pro-fibrinolytic effect of rhAPC, and our results support this conclusion.

In the present study, we did not find an effect of rhAPC on cytokine levels. Levels of IL-6 and IL-10 gradually declined to normal in the course of 2 days and the level of TNF-alpha remained unchanged over time in both treatment groups. In the PROWESS study, the decrease in IL-6 levels was significantly greater in the rhAPC group as compared with the control group [1]. However, in the post-hoc analysis of the PROWESS data by Dhainaut *et al*., there were no significant differences in IL-6 levels between the rhAPC group and the control group [12]. Moreover, Dhainaut *et al*. did not find any difference in levels of TNF-alpha and IL-10 between the two treatment groups. Our findings confirm these results. Dhainaut *et al*. conclude that their results do not provide a strong basis for a systemic anti-inflammatory effect of rhAPC *in vivo *at the therapeutic dose used. Our results support this conclusion. Indeed, the anti-inflammatory effect of rhAPC has only been demonstrated *in vitro *to date [15], using rhAPC concentrations 100- to 1000-fold the concentration achieved in therapeutic circumstances [16,17].

In the present study, no difference in outcome was found between the rhAPC group and the control group, which is probably due to the small number of patients. The numbers of serious adverse events did not differ between groups.

## Conclusion

This study demonstrates that rhAPC resets sepsis-induced thrombin generation within the first 8 h of infusion, without influencing parameters of fibrinolysis and inflammation.

## Key messages

• 		Recombinant human activated protein C resets thrombin generation within the first 8 h of infusion.

• 		The administration of recombinant activated protein C does not influence parameters of fibrinolysis and inflammation.

## Abbreviations

APC = activated protein C; APTT = activated partial thromboplastin time; COPD = chronic obstructive pulmonary disease; EDTA = ethylene diamine tetraacetic acid; ELISA = enzyme-linked immunosorbent assay; ENHANCE = extended evaluation of recombinant activated protein C; EPCR = endothelial protein C receptor; F1+2 = prothrombin fragment F1+2; ICU = intensive care unit; IL = interleukin; NS = not significant; PAI-1 = plasminogen activator inhibitor type 1; PAP = plasmin-antiplasmin complexes; PAR-1 = protease activated receptor type 1; PCI = protein C inhibitor; PROWESS = protein C worldwide evaluation in severe sepsis; PT = prothrombin time; rhAPC = recombinant human activated protein C; SD = standard deviation; SOFA = sepsis-related organ failure assessment ; TAFI = thrombin-activatable fibrinolysis inhibitor; TAT = thrombin-antithrombin complexes; TNF = tumor necrosis factor.

## Competing interests

The authors declare that they have no competing interests.

## Authors' contributions

ACJMdP carried out the data collection and drafted the manuscript. KB and JCMM were responsible for the laboratory analysis. BAH performed the statistical analysis. EdJ and MBV participated in the coordination of the study. HB participated in the study design and helped to draft the manuscript. ML conceived the study, created the design and helped to draft the manuscript. All authors read and approved the final manuscript.

## Figures and Tables

**Table 1:** Baseline characteristics of the patients

Characteristic	rhAPC group (*n *= 12)	Control group (*n *= 12)
Age, years	57 ± 16	66 ± 14
Male sex (%)	7 (58)	7 (58)
Pre-existing conditions, *n*	7	16
Malignancy	2	7
Diabetes	1	3
Hypertension	2	2
COPD	1	2
Myocardial infarction	0	1
Pancreatitis	1	1
Recent surgery (%)	3 (25)	3 (25)
APACHE II score	21 ± 6	22 ± 6
Mechanical ventilation	12	11
Shock	12	12
Vasopressor use	11	11
Renal replacement therapy	4	4
Number of dysfunctional organ systems	4 ± 1	3 ± 1
SOFA score at inclusion	10 ± 3	9 ± 2
Site of infection, *n*		
Lung	8	8
Urinary tract	2	0
Other	2	4
Causes of infection, *n*		
Gram positive	6	4
Gram negative	7	5
Anaerobes	0	1
Fungi	1	2

Data represent mean ± SD. APACHE, Acute Physiology and Chronic Health Evaluation; COPD, chronic obstructive pulmonary disease; SOFA, sepsis-related organ failure assessment.

**Table 2:** Outcome and adverse events

Characteristic	rhAPC group (*n *= 12)	Control group (*n *= 12)	*p *value
28-day survivors, *n*	10	9	1.00
Length of ICU stay, days	15 ± 14	20 ± 15	0.41
Length of hospital stay, days,	35 ± 20	46 ± 41	0.38
Patients discharged home (%)	5 (41.7)	5 (41.7)	1.00
At least one adverse event, *n*	9	7	0.41
Serious bleeding event, *n*	1	0	0.33

Data represent mean ± SD.
